# Prognostic value of the pre-operative serum albumin to globulin ratio in patients with non-metastatic prostate cancer undergoing radical prostatectomy

**DOI:** 10.1007/s10147-021-01952-6

**Published:** 2021-06-28

**Authors:** Abdulmajeed Aydh, Keiichiro Mori, David D’Andrea, Reza Sari Motlagh, Mohammad Abufaraj, Benjamin Pradere, Hadi Mostafaei, Ekaterina Laukhtina, Fahad Quhal, Pierre I. Karakiewicz, Stefano Luzzago, Alberto Briganti, Quoc-Dien Trinh, Mehdi Kardoust Parizi, Derya Tilki, Dmitry V. Enikeev, Shahrokh F. Shariat

**Affiliations:** 1 Department of Urology, Comprehensive Cancer Center, Vienna General Hospital, Medical University of Vienna, Währinger Gürtel 18-20, 1090 Vienna, Austria; 2 Department of Urology, King Faisal Medical City, Abha, Saudi Arabia; 3 Department of Urology, Jikei University School of Medicine, Tokyo, Japan; 4 Division of Urology, Department of Special Surgery, Jordan University Hospital, The University of Jordan, Amman, Jordan; 5 Department of Urology, University Hospital of Tours, Tours, France; 6 Research Center for Evidence Based Medicine, Tabriz University of Medical Sciences, Tabriz, Iran; 7 Institute for Urology and Reproductive Health, Sechenov University, Moscow, Russia; 8 Department of Urology, King Fahad Specialist Hospital, Dammam, Saudi Arabia; 9 Cancer Prognostics and Health Outcomes Unit, Division of Urology, University of Montreal Health Center, Montreal, Canada; 10 Department of Urology, IRCCS European Institute of Oncology (IEO), Milan, Italy; 11 Division of Oncology/Unit of Urology, URI, IRCCS Ospedale San Raffaele, Milan, Italy; 12 Division of Urological Surgery, Brigham and Women’s Hospital, Harvard Medical School, Boston, MA USA; 13 Department of Urology, Shariati Hospital, Tehran University of Medical Sciences, Teheran, Iran; 14 Martini-Klinik Prostate Cancer Center, Department of Urology, University Hospital Hamburg-Eppendorf, Hamburg, Germany; 15 Departments of Urology, Weill Cornell Medical College, New York, NY USA; 16 Department of Urology, University of Texas Southwestern, Dallas, TX USA; 17 Department of Urology, Second Faculty of Medicine, Charles University, Prague, Czech Republic; 18 European Association of Urology Research Foundation, Arnhem, Netherlands; 19 Karl Landsteiner Institute, Vienna, Austria

**Keywords:** Albumin, Globulin, Radical prostatectomy, Prostate cancer

## Abstract

**Purpose:**

To evaluate the potential predictive value of the preoperative serum albumin to globulin ratio (AGR) for oncological outcomes in patients treated with radical prostatectomy (RP) for clinically non-metastatic prostate cancer (PCa).

**Methods:**

Pre-operative AGR was assessed in a multi-institutional cohort of 6041 patients treated with RP. Logistic regression analyses were performed to assess the association of the AGR with advanced disease. We performed Cox regression analyses to determine the relationship between AGR and biochemical recurrence (BCR).

**Results:**

The optimal cut-off value was determined to be 1.31 according to receiver operating curve analysis. Compared to patients with a higher AGR, those with a lower preoperative AGR had worse BCR-free survival (*P* < 0.01) in the Kaplan–Meier analysis. Pre- and post-operative multivariable models that adjusted for the effects of established clinicopathologic features, confirmed its independent association with BCR [hazard ratio (HR) 1.52, 95% confidence interval (CI) 1.31–1.75, *P *< 0.01, HR 1.55, 95% CI 1.34–1.79, *P *< 0.01, respectively]. However, the addition of AGR to established prognostic models did not improve their discrimination.

**Conclusion:**

While AGR is significantly associated with BCR, in the present study, the clinical impact of AGR was not large enough to affect patient management. Longer follow-up is necessary to observe the true effect of AGR.

## Introduction

Prostate cancer (PCa) is estimated to be the most commonly diagnosed cancer in men and the second leading cause of cancer-related deaths in the United States in 2020 [1]. While there are several treatment options for PCa depending on the risk stratification, radical prostatectomy (RP) is currently the most common treatment for patients with clinically non-metastatic PCa who have long life expectancy [2–5]. However, despite adequate surgery, a significant proportion of patients experience disease recurrence and progression due to clinically occult micrometastases and underestimating tumor aggressiveness [6–9].

Chronic inflammation plays a vital role in carcinogenesis and progression. Inflammatory mediators such as cytokines, chemokines, growth factors, prostaglandins, reactive oxygen, and nitrogen species have been shown to exhibit biomarker potential for PCa [6, 7, 9]. Although clinical parameters such as prostate-specific antigen (PSA), imaging, and Gleason score allow certain risk stratification, they remain suboptimal for staging and prognostication [5]. Preoperative biomarkers could offer a personalized treatment approach for patients. However, preoperative biomarkers that can predict either treatment response or other oncological outcomes in patients with non-metastatic PCa lack standardization, as they need to be better than what we have while remaining simple and cost-effective [10–12]. Among these biomarkers, is the serum albumin to globulin ratio (AGR); in which albumin reflects the body’s nutritional status and globulin reflects the immunological status through its roles in immunity and inflammation [13]. Several studies have shown an inverse association between blood-based AGR and different cancer prognoses [14–16]. To date, the staging and prognostic value of noninvasive AGR have not yet been investigated in patients with non-metastatic PCa.

This study aimed to assess whether preoperative serum AGR could be a reliable biomarker of oncological outcomes in patients undergoing RP for non-metastatic PCa. We hypothesized that preoperative serum AGR could predict outcomes after RP with significant accuracy.

## Materials and methods

### Patient selection

We performed a retrospective analysis of patients treated with RP from our multi-institutional database. Between 2000 and 2011, a total of 6,041 patients with clinically non-metastatic PCa were identified. Due to the retrospective nature of the study, the preoperative staging was not standardized. Non-metastatic disease was defined as no cancer spread from the primary site to different sites in the body. All patients did not receive preoperative or post-operative adjuvant hormonal and radiation therapy. The local ethics committees approved the study at all institutions.

### Intervention

According to the guideline recommendations at the time of recruitement and the surgeon discretion, all patients were treated by RP with or without pelvic lymph node dissection. Dedicated genitourinary pathologists analyzed the specimens at each center. The pathologic stage and grade were assigned using the 2009 American Joint Committee on Cancer TNM staging system and the International Society of Urological Pathology (ISUP) 2014. Lymphovascular invasion (LVI) was defined as the unequivocal presence of tumor cells within an endothelium-lined space without underlying muscular walls [17].

Preoperative AGR was calculated as ${\text{AGR}}\; = \;{\text{albumin}}/\left( {{\text{total}}\;{\text{protein}}\; - \;{\text{albumin}}} \right)$ and assessed within 30 days before RP as part of the preoperative workup.

### Follow-up

Due to the retrospective nature of the study, the follow-up was not standardized. Patients were generally followed by physical examination and PSA measurements taken every three months in the first year of surgery, every six months from the 2nd to 5th year and annually after that. The definition of biochemical recurrence (BCR) was two consecutive PSA readings of more than 0.2 ng/ml [18]. The date of the first rise was considered as the date of BCR. The time to event was calculated from the date of RP to the date of BCR.

### Statistical analyses

The chi-squared test and the Mann–Whitney *U* test were used to compare the distribution of categorical and continuous variables between patients with preoperative AGR > 1.31 and AGR ≤ 1.31, respectively. Cox regression analysis was used to investigate the association of preoperative AGR with BCR-free survival. Kaplan–Meier curves were used to estimate the survival function visually. Two multivariable Cox regression models, including pre- and post-operative clinicopathologic features, were built. The discrimination of these models was assessed using Harrel’s concordance index (C-index). On exploratory analyses, logistic regression modeling was used to investigate preoperative AGR association with lymph node metastasis, positive surgical margin, LVI, and non-organ confined disease (NOCD), defined as ≥ pT3 and/or N + disease. If the 2-sided *P* value was < 0.05, we considered the results to be significant. Data analyses were performed using R (R project, Vienna, Austria).

## Results

### Identification of the optimal cut-off value and association with clinicopathologic features

The preoperative AGR cut-off value was determined by receiver operating characteristics curve analysis using the Youden index [19]. The optimal cut-off in our cohort was 1.31. Using the identified cut-off value, 4038 patients (67%) had an AGR > 1.31 and 2003 (33%) had an AGR ≤ 1.31. Patients characteristics are shown in (Table1). There were no significant differences in clinicopathologic features between patients with AGR > 1.31 and AGR ≤ 1.31 (all *P *> 0.05).

**Table 1:** Clinicopathologic characteristics of 6,041patients treated with Radical Prostatectomy for non-metastatic prostate cancer, stratified by pre-operative Albumin-to-Globulin Ration (AGR)

Variables	Total	Normal AGR	Low AGR	*P* value
Number of patients, *n* (%)	6041	4038	2003	
Median age (IQR)	61.00 [57.00, 66.00]	62.00 [57.00, 66.00]	61.00 [57.00, 66.00]	0.87
Biopsy ISUP (%)				
1	3651 (60.44)	2427 (60.10)	1224 (61.11)	0.055
2	1362 (22.55)	899 (22.26)	463 (23.12)	
3	646 (10.69)	451 (11.17)	195 (9.74)	
4	280 (4.63)	201 (4.98)	79 (3.94)	
5	102 (1.69)	60 (1.49)	42 (2.10)	
Total PSA before RP (median [IQR])	6.00 [4.00, 9.00]	6.00 [4.00, 9.00]	6.00 [4.00, 9.00]	0.53
Clinical tumor stage (%)				
cT1	4299 (71.2)	2874 (71.2)	1425 (71.1)	0.87
cT2	1714 (28.4)	1144 (28.3)	570 (28.5)	
cT3	28 (0.5)	20 (0.5)	8 (0.4)	
Blood transfusion (%)	751 (12.4)	484 (12.0)	267 (13.3)	0.14
Pathological ISUP (%)				
1	1932 (32.0)	1282 (31.7)	650 (32.5)	0.58
2	2187 (36.2)	1471 (36.4)	716 (35.7)	
3	1512 (25.0)	1022 (25.3)	490 (24.5)	
4	202 (3.34)	133 (3.29)	69 (3.44)	
5	208 (3.44)	130 (3.22)	78 (3.89)	
Pathological tumor stage (%)				
< = T2	4674 (77.4)	3133 (77.6)	1541 (76.9)	0.74
T3a	1006 (16.7)	670 (16.6)	336 (16.8)	
> = T3b	361 (6.0)	235 (5.8)	126 (6.3)	
LN metastasis (%)				
pN0	2514 (41.6)	1709 (42.3)	805 (40.2)	0.12
pN1	41 (0.68)	31 (0.77)	10 (0.50)	
pNx	3486 (57.7)	2298 (56.9)	1188 (59.3)	
Positive surgical margin (%)	794 (13.1)	541 (13.4)	253 (12.6)	0.41
LVI (%)	693 (11.5)	465 (11.5)	228 (11.4)	0.88

*AGR* Albumin to globulin ratio, *RP* Radical Prostatectomy, *PSA* Prostatic-specific Antigen, *ISUP* International Society of Urological Pathology, *LN metastasis* Lymph Node metastasis, *PSM* positive surgical margin, *LVI* Lymphovascular invasion

### Association with biochemical recurrence

**Fig. 1:**
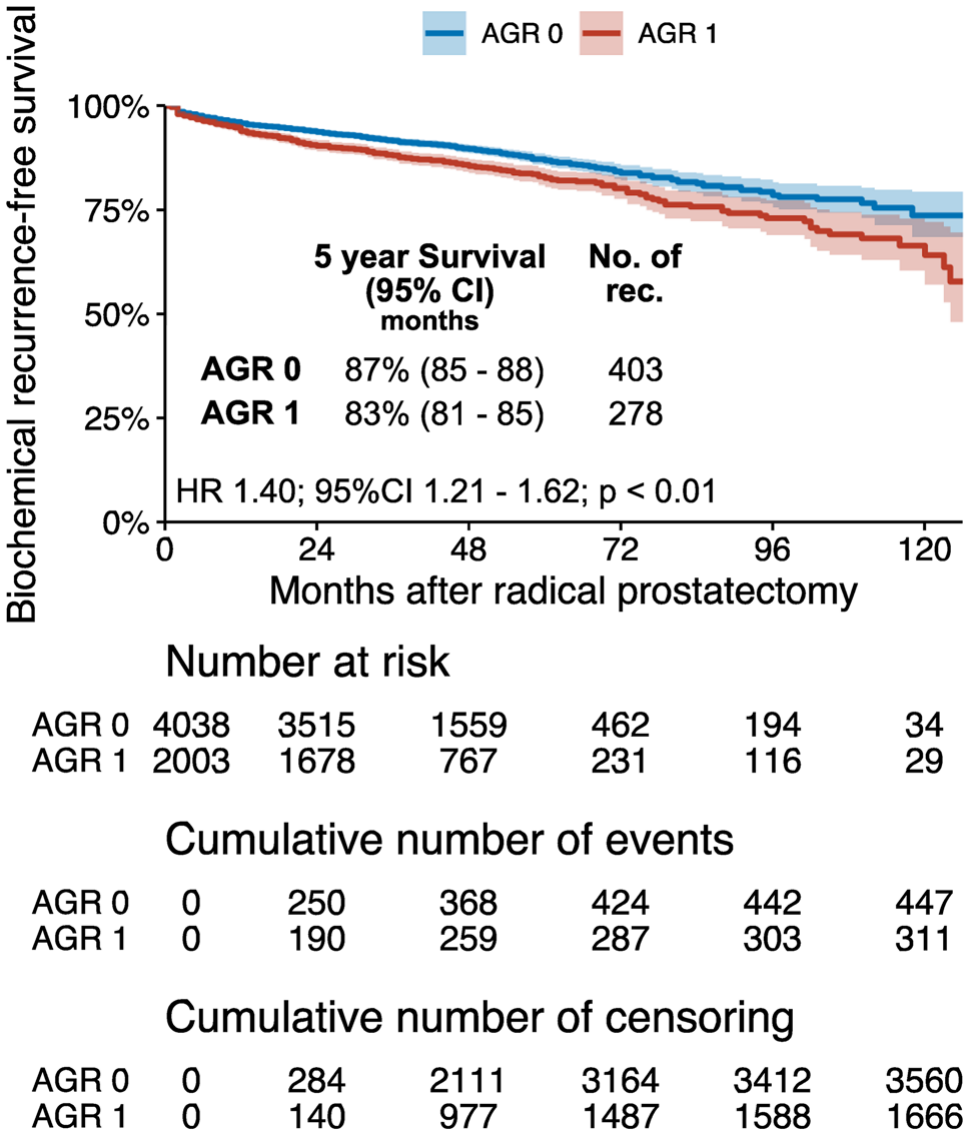
Kaplan–Meier analysis for BCR-free survival in 6041 patients treated with radical prostatectomy for clinically non-metastatic prostate cancer, stratified by AGR. *BCR* Biochemical Recurrence, *RP* radical prostatectomy.

During a median follow-up of 45 months (interquartile range 35–58), 681 patients experienced BCR. In all, 278 (40.8%) had a preoperative AGR ≤ 1.31, and 403 (59.2%) had a preoperative AGR > 1.31. On univariable Cox regression analysis, preoperative AGR ≤ 1.31 was associated with a higher risk of BCR [hazard ratio (HR) 1.40; 95% confidence interval (CI) 1.21–1.62; *P *< 0.01] (Fig. 1).

On multivariable Cox regression analyses that adjusted for preoperative and post-operative variables, AGR ≤ 1.31 remained significantly associated with BCR. The addition of AGR to the base models did not improve their discrimination (Table 2).

**Table 2:** Cox regression analyses for the prediction of biochemical recurrence

Variable	Univariable analysis			Multivariable analysis		
		HR (95% CI)	*P*		HR (95% CI)	*P*
Pre-operative model						
Total PSA before RP	1.05	(1.05–1.06)	< 0.01	1.05	(1.04–1.05)	< 0.01
Biopsy tumor ISUP						
ISUP1	Ref	Ref	Ref			
ISUP2	1.96	(1.63–2.35)	< 0.01	1.87	(1.56–2.25)	< 0.01
ISUP3	3.25	(2.67–3.96)	< 0.01	3.07	(2.52–3.74)	< 0.01
ISUP4	4.77	(3.73–6.09)	< 0.01	4.23	(3.30–5.41)	< 0.01
ISUP5	8.14	(5.88–11.26)	< 0.01	5.78	(4.13–8.11)	< 0.01
Clinical tumor stage						
T2	Ref	Ref	Ref			
T3	7.80	(4.88–12.47)	< 0.01	4.61	(2.86–7.44)	< 0.01
AGR	1.40	(1.21–1.62)	< 0.01	1.50	(1.30–1.74)	< 0.01
Accuracy without AGR	0.7388					
Accuracy with AGR	0.7410					
Post-operative model						
Total PSA before RP	1.05	(1.05–1.06)	< 0.01	1.04	(1.03–1.04)	< 0.01
Positive surgical margin	3.74	(3.21–4.34)	< 0.01	2.02	(1.72–2.37)	< 0.01
Pathological tumor stage						
T2	Ref	Ref	Ref			
T3	5.31	(4.60–6.13)	< 0.01	2.70	(2.29–3.18)	< 0.01
Lymph node metastasis	14.71	(11.73–18.45)	< 0.01	3.52	(2.68–4.62)	< 0.01
Pathological ISUP						
ISUP1	Ref	Ref	Ref			
ISUP2	1.57	(1.24–198)	< 0.01	1.24	(0.97–1.57)	< 0.08
ISUP3	4.05	(3.26–5.03)	< 0.01	2.38	(1.89–2.99)	< 0.01
ISUP4	9.33	(7.05–12.34)	< 0.01	3.43	(2.53–4.64)	< 0.01
ISUP5	13.72	(10.44–18.04)	< 0.01	3.47	(2.51–4.80)	< 0.01
AGR	1.40	(1.21–1.62)	< 0.01	1.58	(1.36–1.83)	< 0.01
Accuracy without AGR	0.8124					
Accuracy with AGR	0.8164					

*AGR* Albumin to globulin ratio, *PSA* Prostatic Specific Antigen, *ISUP* International Society of Urological Patho

### Association of AGR with perioperative outcomes

Preoperative AGR was not associated with positive surgical margin, LVI, lymph node metastasis, or NOCD (all *P *≥ 0.4) on exploratory logistic regression analyses. (Table 3).

**Table 3:** Logistic regression analyses for the prediction of outcomes

Variable	Positive surgical margin						LVI						Lymph node metastasis						NOCD					
	Univariable analysis			Multivariable analysis			Univariable analysis			Multivariable analysis			Univariable analysis			Multivariable analysis			Univariable analysis			Multivariable analysis		
		OR (95%CI)	*P*		OR (95%CI)	P		OR (95% CI)	*P*		OR (95% CI)	*P*		OR (95% CI)	*P*		OR (95% CI)	*P*		OR (95% CI)	*P*		OR (95%CI)	*P*
PSA	1.06	(1.05–1.08)	< 0.01	1.05	(1.04–1.06)	< 0.01	1.01	(0.99–1.02)	0.10	1.00	(0.99–1.02)	0.45	1.06	(1.04–1.07)	< 0.01	1.02	(0.99–1.04)	0.07	1.09	(1.07–1.09)	< 0.01	1.06	(1.05–1.08)	< 0.01
Pathological ISUP	1.56	(1.45–167)	< 0.01	1.48	(1.38–1.59)	< 0.01	1.21	(1.12–1.30)	< 0.01	1.20	(1.11–1.30)	0.00	5.28	(4.37–6.38)	< 0.01	5.22	(4.30–6.32)	< 0.01	2.89	(2.69–3.11)	< 0.01	2.77	(2.57–2.98)	< 0.01
AGR	0.93	(0.80–1.10)	0.40	0.93	(0.79–1.09)	0.36	0.99	(0.83–1.17)	0.83	0.98	(0.83–1.16)	0.86	0.83	(0.56–1.25)	0.37	0.71	(0.46–1.12)	0.14	1.02	(0.90–1.17)	0.65	1.01	(0.88–1.17)	0.87

*PSA* Prostatic Specific Antigen, *ISUP* International Society of Urological Pathology, *AGR* Albumin to globulin ratio, *LVI* Lymphovascular invasion, *LN* Lymph Node metastasis, *NOCD* Non-organ confined disease

We also performed a sub-group analysis according to the European Association of Urology (EAU) risk group classification [20]. We found that the AGR status did not show an association between AGR and adverse perioperative features (all *P* value > 0.05).

## Discussion

To our knowledge, this is the first study to evaluate preoperative serum AGR as a biomarker to predict BCR and oncological outcomes after primary RP for localized prostate cancer patients. Emerging evidence has shown that AGR could predict cancer diagnosis and prognosis in several malignancies, including colorectal [21], gastric [22], lung [23], and breast [24]. Our results demonstrated that low AGR was significantly associated with the risk of BCR in patients with localized prostate cancer undergoing RP. One of the factors that may explain this association is the increase in the concentration of free testosterone secondary to the low albumin-bound testosterone, which eventually can influence disease recurrence and progression. Moreover, the consequence of inflammatory mediators during systematic inflammation can also be associated with tumor progression [25]. Indeed, it is well-known that inflammation has an essential role in tumor progression.

In our study, none of the biological aggressiveness indicators were correlated with low AGR level. Therefore, the precise mechanism in which AGR can influence BCR is still unknown. Notably, we assessed if the AGR level could predict lymph node metastasis, LVI, positive surgical margin, or NOCD. A low preoperative AGR was not found to be correlated with any of these outcomes. A possible explanation of why AGR may not be associated with perioperative outcomes in PCa is that patients chosen for RP as a treatment are presumed to be healthy with no significant comorbidities, and they are also presumed to have localized disease. This is contrary to other malignancies and to advanced PCa patients who could be offered hormonal or radiation treatment. Besides, due to PSA screening, the disease is detected in an early stage.

While low preoperative serum AGR was not associated with aggressive disease features such as pathological Gleason score and LN metastasis, the association with BCR could be important for decision making based on prognostic risk estimations. Despite the promising role of this biomarker in our study, only one study has evaluated the association of AGR in patients with metastatic PCa receiving androgen deprivation therapy and showed that a low serum AGR was an independent predictor of progression and cancer-specific mortality [26]. Because of the literature paucity, further studies should investigate AGR role in different stages of PCa to validate this conclusion.

Several limitations of the present study should be taken into consideration. The main limitation is the retrospective design and multicentric nature of this study. In addition, one of the major limitations of the study is the short follow-up. Another limitation is the lack of standardization of clinical staging for patients. Furthermore, as this is a multicentric study, the surgeries were performed by different surgeons and the RP specimens were analyzed in different laboratories. Moreover, we could not investigate the overall survival and cancer-specific survival because of the lack of mortality data. Despite these limitations, we provided the first reliable study to evaluate the AGR as a biomarker in patients with non-metastatic PCa patients who underwent RP.

## Conclusion

While AGR is significantly associated with BCR, in the present study, the clinical impact of AGR was not large enough to affect patient management. Further studies with longer follow-up are necessary to further understand the prognostic impact of AGR in patients with prostate cancer.

## References

[1] Siegel RL, Miller KD, Jemal A (2020). Cancer statistics, 2020. CA Cancer J Clin.

[2] Walz J (2007). Clinicians are poor raters of life-expectancy before radical prostatectomy or definitive radiotherapy for localized prostate cancer. BJU Int.

[3] Lughezzani G (2010). Predictive and prognostic models in radical prostatectomy candidates: a critical analysis of the literature. Eur Urol.

[4] Gallina A (2008). Comparison of stage migration patterns between Europe and the USA: an analysis of 11 350 men treated with radical prostatectomy for prostate cancer. BJU Int.

[5] Shariat SF (2009). Critical review of prostate cancer predictive tools. Future Oncol.

[6] Shariat SF (2011). Tumor markers in prostate cancer I: blood-based markers. Acta Oncol.

[7] Karam JA (2007). Caveolin-1 overexpression is associated with aggressive prostate cancer recurrence. Prostate.

[8] Chun FK (2006). Significant upgrading affects a third of men diagnosed with prostate cancer: predictive nomogram and internal validation. BJU Int.

[9] Shariat SF (2004). Association of pre-operative plasma levels of vascular endothelial growth factor and soluble vascular cell adhesion molecule-1 with lymph node status and biochemical progression after radical prostatectomy. J Clin Oncol.

[10] Bensalah K, Montorsi F, Shariat SF (2007). Challenges of cancer biomarker profiling. Eur Urol.

[11] Shariat SF (2010). Statistical consideration for clinical biomarker research in bladder cancer. Urol Oncol.

[12] Shariat SF (2008). Improved prediction of disease relapse after radical prostatectomy through a panel of pre-operative blood-based biomarkers. Clin Cancer Res.

[13] Gabay C, Kushner I (1999). Acute-phase proteins and other systemic responses to inflammation. N Engl J Med.

[14] Du XJ (2014). The pretreatment albumin to globulin ratio has predictive value for long-term mortality in nasopharyngeal carcinoma. PLoS ONE.

[15] Li J (2020). Prognostic value of pretreatment albumin to globulin ratio in lung cancer: a meta-analysis. Nutr Cancer.

[16] Shibutani M (2015). The pretreatment albumin to globulin ratio predicts chemotherapeutic outcomes in patients with unresectable metastatic colorectal cancer. BMC Cancer.

[17] Shariat SF (2004). Lymphovascular invasion is a pathological feature of biologically aggressive disease in patients treated with radical prostatectomy. J Urol.

[18] Stephenson AJ (2006). Defining biochemical recurrence of prostate cancer after radical prostatectomy: a proposal for a standardized definition. J Clin Oncol.

[19] Youden WJ (1950). Index for rating diagnostic tests. Cancer.

[20] European Association, U (2020). European association of urology guidelines. 2020 edition. Vol. presented at the EAU annual congress Amsterdam 2020.

[21] Azab B (2013). The value of the pretreatment albumin/globulin ratio in predicting the long-term survival in colorectal cancer. Int J Colorectal Dis.

[22] Mao M-J (2017). Clinical significance of pre-operative albumin and globulin ratio in patients with gastric cancer undergoing treatment. Biomed Res Int.

[23] Duran AO (2014). Albumin-globulin ratio for prediction of long-term mortality in lung adenocarcinoma patients. Asian Pac J Cancer Prev: APJCP.

[24] Azab BN (2013). Value of the pretreatment albumin to globulin ratio in predicting long-term mortality in breast cancer patients. Am J Surg.

[25] Coussens LM, Werb Z (2002). Inflammation and cancer. Nature.

[26] Wang N (2018). Pretreatment serum albumin/globulin ratio as a prognostic biomarker in metastatic prostate cancer patients treated with maximal androgen blockade. Asian J Androl.

